# The hemochromatosis protein HFE signals predominantly via the BMP type I receptor ALK3 in vivo

**DOI:** 10.1038/s42003-018-0071-1

**Published:** 2018-06-08

**Authors:** Lisa Traeger, Caroline A. Enns, Jan Krijt, Andrea U. Steinbicker

**Affiliations:** 1 Department of Anesthesiology, Intensive Care and Pain Medicine, University Hospital Muenster, University of Muenster, Albert-Schweitzer Campus 1, 48149 Muenster, Germany; 2 Department of Cell, Developmental, and Cancer Biology, Oregon Health & Science University, 3181 SW Sam Jackson Park Road, Portland, OR 97239 USA; 3 Institute of Pathological Physiology, First Faculty of Medicine, Charles University, U Nemocnice 5, Prague, 128 53 Czech Republic

## Abstract

Mutations in *HFE*, the most common cause of hereditary hemochromatosis, lead to iron overload. The iron overload is characterized by increased iron uptake due to lower levels of the hepatic, iron regulatory hormone hepcidin. *HFE* was cloned 21 years ago, but the signaling pathway is still unknown. Because bone morphogenetic protein (BMP) signaling is impaired in patients with hereditary hemochromatosis, and the interaction of HFE and the BMP type I receptor ALK3 was suggested in vitro, in vivo experiments were performed. In vivo, hepatocyte-specific *Alk3*-deficient and control mice were injected with either AAV2/8-*Hfe-Flag* or PBS. HFE overexpression in control mice results in increased hepatic hepcidin levels, p-Smad1/5 levels, and iron deficiency anemia, whereas overexpression of HFE in hepatocyte-specific *Alk3*-deficient mice results in no change in hepcidin, p-Smad1/5 levels, or blood parameters. These results indicate that HFE signals predominantly via ALK3 to induce hepcidin in vivo.

## Abstract (web summary)

Lisa Traeger et al. show that human hereditary hemochromatosis protein (HFE) signals through ALK3 to induce hepcidin in mice in vivo. The results validate and extend previous findings from in vitro studies that suggested a link between HFE and BMP signaling.

## Introduction

Hereditary hemochromatosis is the most frequent, inherited autosomal recessive disorder, with an allele frequency of 1 in 8 in people of Northern European descent. Iron overload occurs as a result of a deficiency in the expression of hepcidin. Hepcidin binds to the sole known iron exporter, ferroportin. Upon binding, ferroportin is ubiquitinated and degraded, which results in the inhibition of iron absorption from the diet and of iron release from macrophages^1–3^. In hereditary hemochromatosis, the regulation of hepcidin expression is impaired, so that iron uptake is increased^4–7^. Iron overload occurs because vertebrates have no mechanisms to control iron efflux from their bodies.

Iron toxicity presents predominantly as liver failure, cardiomyopathy, and diabetes. So far, treatment has been limited to repeated phlebotomy or administration of iron chelators. However, given that symptoms of hereditary hemochromatosis are typically non-specific and iron accumulation gradual, the disease often remains undiagnosed for decades^4,5^.

Primary hereditary hemochromatosis is most commonly caused by mutations in *HFE*, encoding the human hereditary hemochromatosis protein (HFE), thus causing iron overload^8^. Humans with iron overload-causing mutations and mice lacking *Hfe* have lower expression of hepcidin for the amount of iron in the body, leading to the hypothesis that the human mutations are caused by loss of function of HFE^5,9^. Mutations in other genes such as transferrin receptor 2 (TfR2), hemojuvelin, hepcidin, and BMP6 can cause rare forms of hereditary hemochromatosis^5,6^.

In vitro studies suggest that HFE is part of an iron-sensing complex composed of HFE, TfR1, and TfR2. The complex regulates hepcidin expression in response to iron-loaded transferrin (holo-transferrin). A current model hypothesizes that at low transferrin saturation, TfR1 sequesters HFE. Tf binding to TfR1 competes with and releases HFE to interact with TfR2. The HFE–TfR2 complex positively regulates hepcidin expression^10,11^. This model remains controversial, as no direct interaction between TfR2 and HFE has been detected in vivo^12,13^. The signaling pathway used by either HFE alone or the TfR2–HFE complex to induce hepcidin expression has yet to be elucidated. In addition to TfR2, HFE requires HJV to induce hepcidin regulation and was shown to form a multi-protein complex with TfR2 and HJV at the cell surface of Huh7 cells in vitro^14,15^. Previous studies suggested that HFE may regulate hepcidin expression through the BMP pathway: the characterization of mice with *Hfe* deficiency revealed that BMP/Smad signaling was impaired in these mice^16^, but the definitive in vivo evidence is lacking.

Twenty one years after the discovery of HFE, this study demonstrates that HFE failed to stimulate hepcidin expression in the liver in the absence of the BMP type I receptor ALK3 in vivo in mice. The results confirm the former in vitro experiments, extend the findings, and provide evidence that HFE acts through the BMP signaling pathway, namely ALK3, to control hepcidin expression.

## Results

### HFE interacts with ALK3 but not with ALK2 in vitro

HFE could affect BMP signaling by directly interacting with the BMP type I receptor. Previous studies demonstrated that ALK3 and to a lesser extent ALK2 were critical to maintain iron homeostasis in mice^18^. Wu et al. showed that HFE co-precipitated with ALK3 suggesting that ALK3 interacts with HFE in vitro^17^. We performed co-immunoprecipitation of tagged receptors and confirmed the interaction of ALK3 with HFE in vitro (Supplementary Fig. 1a). Because the BMP type I receptor ALK2 is also expressed in the liver and is required for optimal hepcidin induction^18^, co-immunoprecipitation of HFE with ALK2 was performed. HFE failed to co-immunoprecipitate with ALK2 in Huh7 cells (Supplementary Fig. 1b). These results indicate that ALK3, but not ALK2, does detectably interact with HFE in vitro.

### HFE is overexpressed in mice injected with AAV2/8-HFE-Flag

**Fig. 1:**
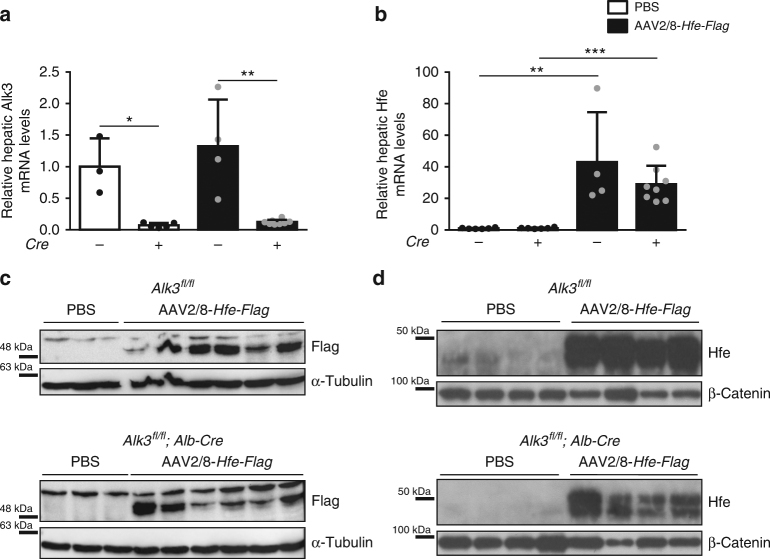
Mice injected with the AAV2/8-*Hfe-Flag* overexpressed HFE 14 days after virus administration. Hepatocyte-specific *Alk3-*deficient male mice as well as control mice were injected with 5*10^11^ particles of AAV2/8-*Hfe-Flag* and analyzed after 14 days. **a** *Alk3*^*fl/fl*^*; Alb-Cre* mice had significantly lower levels of Alk3 mRNA compared to *Alk3*^*fl/fl*^ mice (*Alk3*^*fl/fl*^*: n* = 3; *Alk3*^*fl/fl*^*; Alb-Cre*: *n* = 5; *Alk3*^*fl/fl*^ injected with AAV2/8-*Hfe-Flag*: *n* = 4; *Alk3*^*fl/fl*^*; Alb-Cre* injected with AAV2/8-*Hfe-Flag*: *n* = 8; *p = 0.0375, **p = 0.004). **b** Hfe mRNA levels increased in mice with and without hepatocyte-specific *Alk3* deficiency injected with AAV2/8-*Hfe-Flag* compared to appropriate control mice injected with PBS (*Alk3*^*fl/fl*^*: n* = 6; *Alk3*^*fl/fl*^*; Alb-Cre*: *n* = 6; *Alk3*^*fl/fl*^ injected with AAV2/8-*Hfe-Flag*: *n* = 4; *Alk3*^*fl/fl*^*; Alb-Cre* injected with AAV2/8-*Hfe-Flag*: *n* = 8; **p = 0.0095; ***p = 0.0007). **c** Hepatic expression of the “Flag” domain of the HFE-Flag is shown. α-tubulin was used as a loading control. The Flag-tagged HFE protein was detected in liver samples of mice with and without hepatocyte-specific *Alk3* deficiency injected with AAV2/8-*Hfe-Flag*. **d** Hepatic HFE protein expression of plasma-enriched membrane fractions is shown. Beta-Catenin was used as a loading control. HFE levels were increased in mice with and without hepatocyte-specific *Alk3* deficiency injected with AAV2/8-*Hfe-Flag* compared to controls injected with PBS

To address whether the effect of HFE on hepcidin expression is dependent on the expression of ALK3 in vivo, mice with hepatocyte-specific *Alk3* deficiency (*Alk3*^*fl/fl*^*; Alb-Cre*^18^) and their appropriate controls (*Alk3*^*fl/*fl^ mice) were injected with an adeno-associated virus (AAV) encoding *Hfe-Flag* under the control of a liver-specific promotor (AAV2/8-*Hfe-Flag*) and compared to animals injected with vehicle (PBS). Fourteen days after virus administration, blood and tissues were harvested and analyzed. The absence of inflammation, efficiency of knockout, and effectiveness of *HFE* expression were verified. As previously shown, the AAV2/8 virus itself does not cause an inflammatory response, which could result in the induction of hepcidin mRNA independent of the iron status^19^. Consistently, mice expressed similar levels of IL-6 mRNA (Supplementary Fig. 2a). Hepatocyte-specific *Alk3*-deficient mice presented with a reduction of Alk3 mRNA levels by 90% when compared to control mice (Fig. 1a). In mice with and without hepatocyte-specific *Alk3* deficiency injected with AAV2/8-*Hfe-Flag*, hepatic Hfe mRNA levels were increased to similar extents (Fig. 1b).

HFE-Flag was detected in livers of animals injected with AAV2/8-*Hfe-Flag* (Fig. 1c) and in membrane-enriched fractions of the liver (Fig. 1d, Supplementary Fig. 2b). The data indicate that *Alk3*^*fl/fl*^*; Alb-Cre* mice were deficient for hepatic Alk3 and that all mice injected with AAV2/8-*Hfe-Flag* were successfully overexpressing HFE after 14 days.

### HFE overexpression caused anemia in control mice

**Fig. 2:**
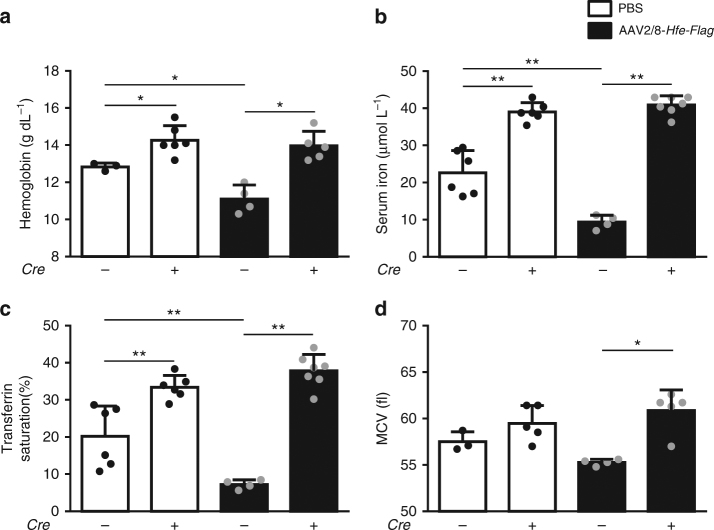
HFE overexpression caused anemia in control mice, but not in mice with hepatocyte-specific *Alk3* deficiency. Hematological and iron parameters were analyzed 14 days after AAV2/8-*Hfe-Flag* or PBS injection in mice with and without hepatocyte-specific *Alk3* deficiency. **a** Hemoglobin levels (*Alk3*^*fl/fl*^*: n* = 3; *Alk3*^*fl/fl*^*; Alb-Cre*: *n* = 6; *Alk3*^*fl/fl*^ injected with AAV2/8-*Hfe-Flag*: *n* = 4; *Alk3*^*fl/fl*^*; Alb-Cre* injected with AAV2/8-*Hfe-Flag*: *n* = 5; *p ≤ 0.0238), **b** serum iron levels (*Alk3*^*fl/fl*^*: n* = 6; *Alk3*^*fl/fl*^*; Alb-Cre*: *n* = 6; *Alk3*^*fl/fl*^ injected with AAV2/8-*Hfe-Flag*: *n* = 4; *Alk3*^*fl/fl*^*; Alb-Cre* injected with AAV2/8-*Hfe-Flag*: *n* = 7; **p ≤ 0.0095), **c** transferrin saturation (*Alk3*^*fl/fl*^*: n* = 6; *Alk3*^*fl/fl*^*; Alb-Cre*: *n* = 6; *Alk3*^*fl/fl*^ injected with AAV2/8-*Hfe-Flag*: *n* = 4; *Alk3*^*fl/fl*^*; Alb-Cre* injected with AAV2/8-*Hfe-Flag*: *n* = 7; **p ≤ 0.0095) and **d** mean corpuscular volume (MCV) (*Alk3*^*fl/fl*^*: n* = 3; *Alk3*^*fl/fl*^*; Alb-Cre*: *n* = 5; *Alk3*^*fl/fl*^ injected with AAV2/8-*Hfe-Flag*: *n* = 4; *Alk3*^*fl/fl*^*; Alb-Cre* injected with AAV2/8-*Hfe-Flag*: *n* = 5; *p = 0.0159) are shown

Increased HFE expression in wild-type (WT) mice results in increased, phosphorylation of Smad 1/5/8, which induces hepcidin expression. The induction of hepcidin in WT mice leads to anemia^14^. We used mice with and without hepatocyte-specific *Alk3* deficiency injected either with AAV2/8-*Hfe-Flag* or PBS to determine whether HFE induction of hepcidin is dependent on ALK3 expression in vivo. Control mice (*Alk3*^*fl/fl*^) injected with AAV2/8-*Hfe-Flag* developed normocytic anemia when compared to PBS-injected animals. Hemoglobin levels, transferrin saturation, and serum iron levels were reduced (Fig. 2a–c). The mean corpuscular volume (MCV) was within a similar range in mice injected with AAV2/8-*Hfe-Flag* compared to PBS-injected controls (Fig. 2d).

Hepatocyte-specific deficiency of the BMP type I receptor *Alk3* (*Alk3*^*fl/fl*^*; Alb-Cre*) causes an imbalance of the systemic iron homeostasis and hence iron overload, as published previously^18^. Mice with hepatocyte-specific *Alk3* deficiency injected with PBS presented with higher hemoglobin levels, serum iron levels, and transferrin saturation, and a similar MCV compared to control mice injected with PBS. In contrast to *Alk3*^*fl/fl*^ mice, mice with hepatocyte-specific *Alk3* deficiency were unresponsive to the overexpression of *Hfe-Flag* as they did not develop anemia or present with a reduced iron status. Hemoglobin levels, serum iron levels, and transferrin saturation remained high when compared to mice with hepatocyte-specific *Alk3* deficiency injected with PBS (Fig. 2a–c). The data indicate that HFE overexpression leads to the development of anemia in control mice. However, HFE overexpression in mice lacking ALK3 showed no changes in serum iron, Tf-saturation or red blood cell analysis indicating the importance of ALK3 expression on the HFE induced changes in iron homeostasis.

To further test the relationship of HFE and ALK3 in controlling iron homeostasis, non-heme tissue iron levels were measured.

**Fig. 3:**
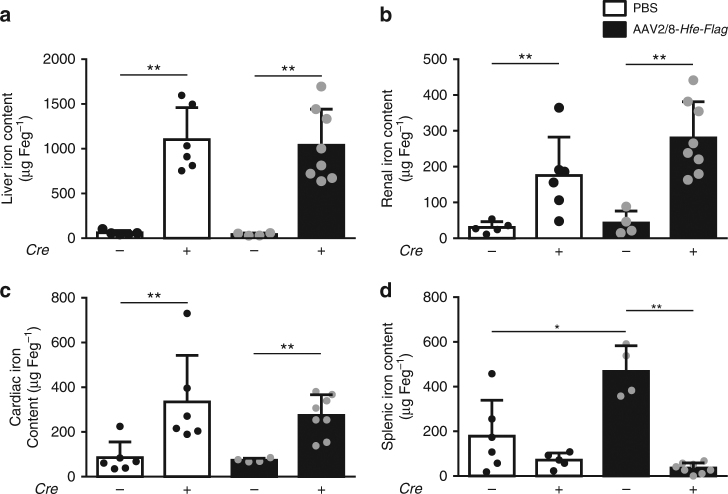
Hepatic, cardiac, and renal iron content remained similar, while splenic iron content increased in control mice overexpressing HFE. Tissue iron content was determined 14 days after virus or vehicle administration. **a** Liver iron content (*Alk3*^*fl/fl*^*: n* = 5; *Alk3*^*fl/fl*^*; Alb-Cre*: *n* = 6; *Alk3*^*fl/fl*^ injected with AAV2/8-*Hfe-Flag*: *n* = 4; *Alk3*^*fl/fl*^*; Alb-Cre* injected with AAV2/8-*Hfe-Flag*: *n* = 8; **p ≤ 0.004), **b** renal iron content (*Alk3*^*fl/fl*^*: n* = 5; *Alk3*^*fl/fl*^*; Alb-Cre*: *n* = 6; *Alk3*^*fl/fl*^ injected with AAV2/8-*Hfe-Flag*: *n* = 4; *Alk3*^*fl/fl*^*; Alb-Cre* injected with AAV2/8-*Hfe-Flag*: *n* = 8; **p ≤ 0.009), **c** cardiac iron content (*Alk3*^*fl/fl*^*: n* = 6; *Alk3*^*fl/fl*^*; Alb-Cre*: *n* = 6; *Alk3*^*fl/fl*^ injected with AAV2/8-*Hfe-Flag*: *n* = 4; *Alk3*^*fl/fl*^*; Alb-Cre* injected with AAV2/8-*Hfe-Flag*: *n* = 8; *p = 0.0152; **p = 0.004), and **d** splenic iron content (*Alk3*^*fl/fl*^*: n* = 6; *Alk3*^*fl/fl*^*; Alb-Cre*: *n* = 5; *Alk3*^*fl/fl*^ injected with AAV2/8-*Hfe-Flag*: *n* = 4; *Alk3*^*fl/fl*^*; Alb-Cre* injected with AAV2/8-*Hfe-Flag*: *n* = 8; *p = 0.0381; **p = 0.004) of control mice and heaptocyte-specific *Alk3*-deficient mice injected with AAV2/8-*Hfe-Flag* or PBS are shown

Mice with hepatocyte-specific *Alk3* deficiency developed iron overload as indicated by increased hepatic, renal, and cardiac iron content. Splenic iron content was decreased as expected in states of iron overload (Fig. 3a–d). Overexpression of HFE did not change hepatic, renal, cardiac, or splenic iron content in mice with hepatocyte-specific *Alk3* deficiency. In contrast, control mice injected with AAV2/8-*Hfe-Flag* developed anemia, and, as a consequence, retained more iron in the spleen compared to PBS-injected controls (Fig. 3d).

These data reveal that hepatic, renal, and cardiac tissue iron loading were not affected by HFE overexpression for 2 weeks and that ALK3 is required for HFE-mediated iron regulation.

### ALK3 is required for HFE-mediated hepcidin induction

**Fig. 4:**
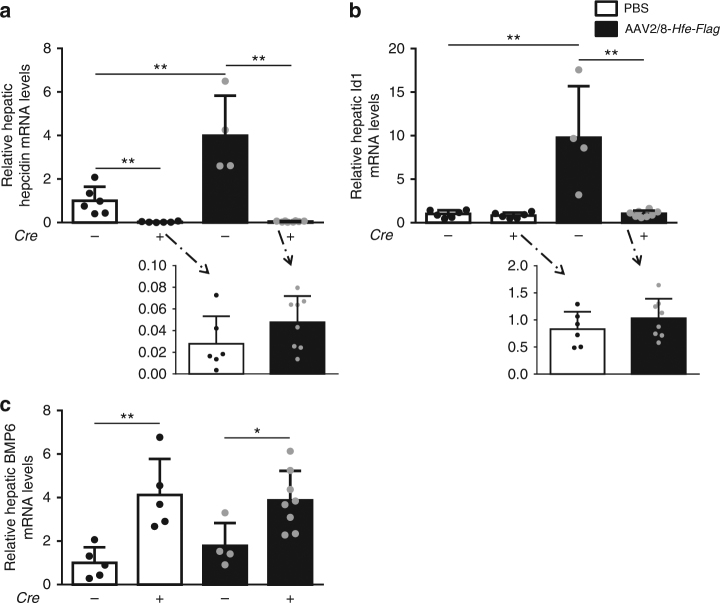
Hepcidin expression was induced by HFE overexpression in control mice. **a** In control animals, overexpression of HFE led to increased hepatic hepcidin mRNA expression, while there was no effect in hepatocyte-specific *Alk3* deficient mice (*Alk3*^*fl/fl*^*: n* = 6; *Alk3*^*fl/fl*^*; Alb-Cre*: *n* = 6; *Alk3*^*fl/fl*^ injected with AAV2/8-*Hfe-Flag*: *n* = 4; *Alk3*^*fl/fl*^*; Alb-Cre* injected with AAV2/8-*Hfe-Flag*: *n* = 8; **p ≤ 0.0095). **b** Hepatic Id1 mRNA levels were upregulated in control mice injected with AAV2/8-*Hfe-Flag* compared to control mice injected with PBS. In contrast, Id1 mRNA levels were not induced by AAV2/8-*Hfe-Flag* injection in mice with hepatocyte-specific *Alk3* deficiency. (*Alk3*^*fl/fl*^*: n* = 6; *Alk3*^*fl/fl*^*; Alb-Cre*: *n* = 6; *Alk3*^*fl/fl*^ injected with AAV2/8-*Hfe-Flag*: *n* = 4; *Alk3*^*fl/fl*^*; Alb-Cre* injected with AAV2/8-*Hfe-Flag*: *n* = 8; **p ≤ 0.0095). **c** Hepatic BMP6 mRNA levels were increased in hepatocyte-specific *Alk3* deficient mice injected with AAV2/8-*Hfe-Flag* or vehicle. (*Alk3*^*fl/fl*^*: n* = 5; *Alk3*^*fl/fl*^*; Alb-Cre*: *n* = 5; *Alk3*^*fl/fl*^ injected with AAV2/8-*Hfe-Flag*: *n* = 4; *Alk3*^*fl/fl*^*; Alb-Cre* injected with AAV2/8-*Hfe-Flag*: *n* = 8; *p = 0.028; **p = 0.008)

Next, hepcidin and Id1 mRNA expression were analyzed. Like hepcidin, Id1 expression is increased by BMP signaling. In control mice injected with AAV2/8-*Hfe-Flag*, hepcidin mRNA expression levels increased fourfold when compared to PBS-injected animals (Fig. 4a). Interestingly, hepcidin mRNA levels in mice with hepatocyte-specific *Alk3* deficiency did not respond to the overexpression of HFE: hepcidin mRNA levels remained low at a level of about 4% of that of the control mice (Fig. 4a, inlay). Id1 expression was increased in control animals overexpressing *Hfe-Flag* compared to PBS-injected animals. The result indicates that HFE overexpression increased BMP signaling. In contrast, hepatocyte-specific *Alk3*-deficient mice overexpressing HFE did not show an increase in *Id1* gene expression (Fig. 4b) indicating that HFE and ALK3 are both required for BMP signaling.

Hepatic BMP6 mRNA levels were increased in hepatocyte-specific *Alk3-*deficient mice due to increased hepatic iron loading. Overexpression of HFE had no impact on BMP6 mRNA levels in control or hepatocyte-specific *Alk3*-deficient mice (Fig. 4c). The data show that HFE does not modulate the effective dose of BMP6 to ALK3.

**Fig. 5:**
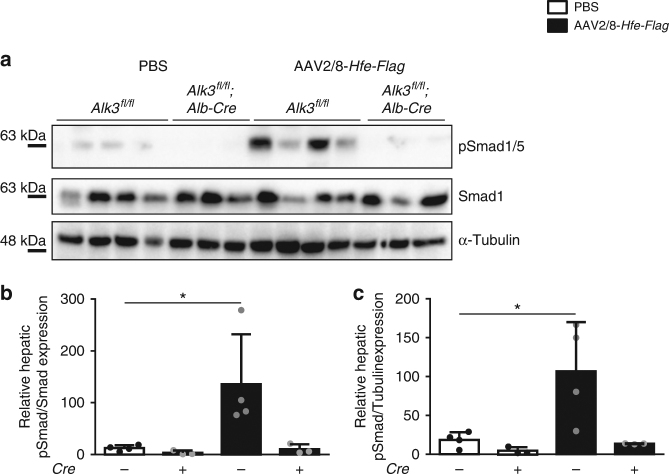
Overexpression of HFE leads to an increase in pSmad1/5 levels in control mice. **a** Hepatic phosphorylation of pSmad1/5, total Smad1 and α-Tubulin protein levels in AAV2/8-*Hfe-Flag*, or PBS-injected mice with and without hepatocyte-specific *Alk3* deficiency are shown. **b** Densitometric analysis of pSMAD1/5/Smad1 of immunoblots depicted in **a** is shown (*Alk3*^*fl/fl*^ mice injected with PBS vs. *Alk3*^*fl/fl*^ mice injected with AAV2/8-*Hfe-Flag*: *p = 0.03; *n* = 3–4). **c** Densitometric analysis of pSMAD1/5/α-Tubulin of immunoblots depicted in **a** is shown (*Alk3*^*fl/fl*^ mice injected with PBS vs. *Alk3*^*fl/fl*^ mice injected with AAV2/8-*Hfe-Flag*: *p = 0.03; *n* = 3–4)

At the protein level, immunoblot analysis revealed that phosphorylated Smad 1/5 levels were increased in control animals overexpressing *Hfe-Flag* compared to animals injected with PBS (Fig. 5a–c, Supplementary Fig. 3a–c). In contrast, hepatocyte-specific *Alk3*-deficient mice did not increase hepatic pSmad1/5 levels after HFE overexpression (Fig. 5a–c, Supplementary Fig. 3a–c). The data indicate that HFE overexpression induced Smad1/5 phosphorylation, hepcidin mRNA expression, and development of anemia.

In contrast, the parameters in mice with a hepatocyte-specific deficiency of *Alk3* remained unchanged indicating the necessity of ALK3 expression for HFE to exert its effects on BMP signaling.

## Discussion

Mutations in the HFE gene reduce hepcidin expression in the liver, thus causing iron overload. Previous studies suggested that HFE may regulate hepcidin expression through the BMP signaling pathway, but the definitive in vivo evidence was lacking. This manuscript demonstrates for the first time to our knowledge that HFE failed to stimulate hepcidin expression by the liver in the absence of the BMP type I receptor ALK3. The results provide unequivocal evidence in vivo that HFE acts through ALK3 to affect the BMP signaling pathway, which controls hepcidin expression.

Other investigators have previously reported an interaction of HFE and the BMP signaling pathway: Kautz and colleagues speculated that HFE and the BMP receptors may be associated at the membrane and required to induce BMP signaling^16^. Phosphorylated Smad, hepcidin, and Id1 levels were reduced in *Hfe-*deficient mice^16^. Studies performed by Corradini et al. postulated that HFE induces hepcidin expression via an interaction with the BMP6–Smad signaling pathway^20,21^. Our study shows the inability of mice with hepatocyte-specific *Alk*3 deficiency to respond to HFE overexpression. The mice are unable to increase hepcidin and Id1 expression or to develop anemia in contrast to control mice. Thus, the HFE-dependent increase in hepcidin expression is dependent on ALK3.

The iron-overload phenotype of mice with hepatocyte-specific *Alk3* deficiency is more severe than the iron-overload phenotype of *Hfe* knockout mice^7,18^, which indicates a dominant role for ALK3. As HFE signals via ALK3, HFE overexpression could not resolve the iron-overload phenotype in mice with hepatocyte-specific *Alk3* deficiency caused by hepcidin deficiency.

How HFE interacts with ALK3 to induce hepcidin in vivo remains to be resolved. Our data indicate that ALK3 and HFE interaction is independent of BMP6, because hepatic BMP6 mRNA levels were not affected by HFE overexpression over this time period. Wu et al. suggested that HFE stabilizes ALK3 at the plasma membrane by preventing its degradation and thereby increasing ALK3 cell surface expression in vitro^17^. In vivo, deficiency of *Hfe* in mice led to a decrease in hepatic ALK3 protein^17^. Another possibility is that HFE links ALK3 to the iron-sensing complex.

TfR2, HFE, and HJV all interact in vitro. HFE could link TfR2 to the HJV/BMP/BMPR/Smad signaling pathway^15^.

In conclusion, the data presented here argues for the first time to our knowledge that the effect that HFE has on hepcidin expression is dependent on ALK3 expression in vivo, and acts through the BMP signaling pathway.

## Methods

### Animals

The current study was performed in accordance with the recommendations and approval of the institutional ethics committee of the North Rhine-Westphalian Agency for Nature, Environment, and Consumer Protection (permit no.: Az. 84-02.04.2014.A015). A Material Transfer Agreement for the use of mice was signed. Mice with homozygous loxP-flanked (“floxed”) *Alk3* alleles (*Alk3*^*fl/fl*^) on a C57BL/6 background with or without a *Cre* recombinase driven by the hepatocyte-specific albumin promotor^18,22,23^ were held in individually ventilated cages and fed a standard diet (198 ppm iron). Eight-week-old male mice with and without a hepatocyte-specific *Alk3* deficiency were injected intravenously with either 5*10^11^ particles of an adeno-associated virus-(AAV2/8) expressing *Hfe-Flag* under the control of a liver-specific promotor (AAV2/8-*Hfe-Flag*^19^) (Vector BioLabs, Malvern, PA, USA) or PBS. PBS was used as a control, as Gao et al. have previously shown that an AAV-expressing GCDH (encoding glutaryl-CoA dehydrogenase, unrelated to iron homeostasis) did not induce hepcidin mRNA expression nor did it affect the iron status^19^.

Two weeks later, euthanasia was performed in deep anesthesia, and blood and organs were collected for analysis.

### Cell culture

Cells from the human hepatocellular carcinoma cell line Huh7 were a kind gift from Martina U. Muckenthaler (Heidelberg, Germany). Cells were maintained in high glucose DMEM (Sigma-Aldrich, Hamburg, Germany) supplemented with 10% FCS (heat inactivated, Capricorn, Ebsdorfergrund, Germany), 2 mM glutamine, 100 units mL^-1^ penicillin, and 100 µg mL^-1^ streptomycin (Sigma-Aldrich, Hamburg, Germany) at 37 °C and 5% CO_2._ Cells were tested negative for mycoplasma contamination (GATC, Konstanz; Germany).

### Hematologic and iron parameters

All efforts were made to minimize suffering. Blood was withdrawn by puncture of the facial vein in deep ketamine/xylazine anesthesia. Complete blood count analysis was performed at the central laboratory of the University Hospital Muenster.

Serum iron concentrations and unsaturated binding capacity (UIBC) were measured using the Iron/UIBC Kit from Biolabo (Maizy, France) according to the manufactures instructions. Non-heme tissue iron levels were determined according to Torrance and Bothwell as described previously^24^.

### Hepatic mRNA levels

RNA was isolated from tissue samples with Trizol® (Sigma-Aldrich, Hamburg, Germany) according to the manufacturer’s instructions.

MMLV-reverse transcriptase (Sigma-Aldrich, Hamburg, Germany) was used to synthesize cDNA. Quantitative RT-PCR was performed on a Bio-Rad CFX Connect™ Real-Time-PCR system using either iTaq™ Universal SYBR® Green Supermix (BioRad, Munich, Germany) or TaqMan Universal Master Mix (Applied Biosystems, Darmstadt, Germany). Primer pairs used for RT-PCR are listed in Supplementary Table 1. Levels of target genes were normalized to 18S levels using the relative C_T_ method.

### Preparation of plasma membrane-enriched fraction

Samples were prepared as previously described^25^. A volume of 200 mg of liver tissue was homogenized in 2.5 mL of 10 mM HEPES with a pH of 7.4 supplemented with 0.25 M sucrose, 5 mM EDTA and a protease inhibitor cocktail (Roche) for 3 × 10 s in a 6 mm Ultra Turrax homogenizer. The homogenate was incubated on ice for 30 min and centrifuged at 400 × *g*, 4 °C, for 10 min. The supernatant was centrifuged at 3000 × *g* for 15 min, the 3000 × *g* pellet was homogenized in 1 mL of 2 M NaCl in10 mM HEPES. Another centrifugation at 3000 × *g* for 15 min was performed. The pellet was again homogenized in 0.1 M sodium carbonate and incubated for 1 h while agitating. After centrifugation at 16,000 × *g* for 1 h the pellet was homogenized in 1 mL of 10 mM HEPES with 4 M urea and incubated on ice for 30 min. The homogenate was centrifuged at 16,000 × *g*. The final pellet was washed with 10 mM HEPES and re-suspended in 125 µL of 25 mM ammonium bicarbonate with 2% SDS. Protein samples were subsequently used for immunoblot analysis.

### Protein analysis

Tissue samples were lysed in RIPA buffer supplemented with protease and phosphatase inhibitors (Sigma-Aldrich, Hamburg, Germany).

Proteins were quantified using the Pierce BCA Protein Assay Kit (Thermo Fisher Scientific, Darmstadt, Germany). An equal amount of proteins was separated by electrophoresis using 4–10% bis–tris gels and blotted on nitrocellulose membranes (GE Healthcare, Freiburg, Germany).

Membranes were incubated overnight with antibodies directed against Flag, c-Myc, α-Tubulin (Sigma-Aldrich, Hamburg, Germany), HFE (Santa-Cruz, Heidelberg, Germany), phosphorylated Smad 5 (Abcam, Cambridge, UK; named pSmad1/5 antibody because of cross-reactivity with pSmad1), total Smad1, E-Cadherin, and β-Catenin (Cell Signaling Technology, Leiden, The Netherlands).

Membranes were washed and incubated with horseradish peroxidase (HRP)-conjugated anti-rabbit- or anti-mouse-IgG (Cell Signaling Technology, Leiden, The Netherlands), and chemiluminescence was detected using ECL-Plus and either the ChemiDoc™ XRS + system (BioRad, Munich, Germany) or Bio-Rad GS 800 scanner (both BioRad, Munich, Germany). Densitometric analysis was performed with the Image Lab software (BioRad, Munich, Germany) or ImageJ. Full uncropped and unedited versions of all immunoblots are depicted in Supplementary Figs. 4–9.

### Plasmids

Eukaryotic expression plasmids encoding ALK2 or ALK3 fused to three copies of the FLAG epitope (3xFLAG-ALK3, 3xFLAG-ALK2; N-terminal) under the control of a CMV promotor were provided by Patricio Leyton and Donald Bloch (Boston, USA). The expression plasmid HFE-cMyc encoding HFE fused to the cMyc epitope at the N-terminus was provided by Martina U. Muckenthaler (Heidelberg, Germany).

### Co-Immunoprecipitation analysis

Huh7 were seeded at a density of 16 × 10^3^ cells per cm^2^. Transfection was performed 16 h later using 15 µg of plasmid DNA and the TransIT®-LT1 transfection reagent (MoBiTec, Goettingen, Germany). Twenty-four hours after transfection, cells were harvested and lysed in NET-buffer. Protein samples were immunoprecipitated using ANTI-FLAG® M2 Affinity Gel (Sigma-Aldrich, Hamburg, Germany) according to the manufactures’ instructions and subsequently used for immunoblot analysis.

### Statistical analysis

Values are expressed at mean ± SD. The corresponding dot plots are overlaid. Data were analyzed with GraphPad Prism (GraphPad Software 6, La Jolla, USA) using non-parametric Mann Whitney *U* tests with a two-tailed *p* value. A *p* value of *p* ≤ 0.05 was considered statistically significant.

### Data availability

The authors confirm that any data not included in the paper and its supplementary files are available from the corresponding author upon request.

## Electronic supplementary material

Supplementary Information

## References

[1] Nemeth E (2004). Hepcidin regulates cellular iron efflux by binding to ferroportin and inducing its internalization. Science.

[2] Pigeon C (2001). A new mouse liver-specific gene, encoding a protein homologous to human antimicrobial peptide hepcidin, is overexpressed during iron overload. J. Biol. Chem..

[3] Hentze MW, Muckenthaler MU, Galy B, Camaschella C (2010). Two to tango: regulation of mammalian iron metabolism. Cell.

[4] Pietrangelo A (2016). Iron and the liver. Liver Int..

[5] Pietrangelo A (2010). Hereditary hemochromatosis: pathogenesis, diagnosis, and treatment. Gastroenterology.

[6] Hollerer I, Bachmann A, Muckenthaler MU (2017). Pathophysiological consequences and benefits of HFE mutations: 20 years of research. Haematologica.

[7] Latour C (2016). Differing impact of the deletion of hemochromatosis-associated molecules HFE and transferrin receptor-2 on the iron phenotype of mice lacking bone morphogenetic protein 6 or hemojuvelin. Hepatology.

[8] Feder JN (1996). A novel MHC class I-like gene is mutated in patients with hereditary haemochromatosis. Nat. Genet..

[9] Steinbicker AU, Muckenthaler MU (2013). Out of balance–systemic iron homeostasis in iron-related disorders. Nutrients.

[10] Feder JN (1998). The hemochromatosis gene product complexes with the transferrin receptor and lowers its affinity for ligand binding. Proc. Natl Acad. Sci. USA.

[11] Schmidt PJ, Toran PT, Giannetti AM, Bjorkman PJ, Andrews NC (2008). The transferrin receptor modulates Hfe-dependent regulation of hepcidin expression. Cell Metab..

[12] Rishi G, Crampton EM, Wallace DF, Subramaniam VN (2013). In situ proximity ligation assays indicate that hemochromatosis proteins Hfe and transferrin receptor 2 (Tfr2) do not interact. PLoS ONE.

[13] Schmidt PJ, Fleming MD (2012). Transgenic HFE-dependent induction of hepcidin in mice does not require transferrin receptor-2. Am. J. Hematol..

[14] Schmidt PJ, Andrews NC, Fleming MD (2010). Hepcidin induction by transgenic overexpression of Hfe does not require the Hfe cytoplasmic tail, but does require hemojuvelin. Blood.

[15] D’Alessio F, Hentze MW, Muckenthaler MU (2012). The hemochromatosis proteins HFE, TfR2, and HJV form a membrane-associated protein complex for hepcidin regulation. J. Hepatol..

[16] Kautz L (2009). BMP/Smad signaling is not enhanced in Hfe-deficient mice despite increased Bmp6 expression. Blood.

[17] Wu XG (2014). HFE interacts with the BMP type I receptor ALK3 to regulate hepcidin expression. Blood.

[18] Steinbicker AU (2011). Perturbation of hepcidin expression by BMP type I receptor deletion induces iron overload in mice. Blood.

[19] Gao J (2010). Hepatocyte-targeted HFE and TFR2 control hepcidin expression in mice. Blood.

[20] Corradini E (2010). BMP6 treatment compensates for the molecular defect and ameliorates hemochromatosis in Hfe knockout mice. Gastroenterology.

[21] Corradini E (2009). Bone morphogenetic protein signaling is impaired in an HFE knockout mouse model of hemochromatosis. Gastroenterology.

[22] Sauer B (1987). Functional expression of the cre-lox site-specific recombination system in the yeast Saccharomyces cerevisiae. Mol. Cell. Biol..

[23] Mishina Y, Hanks MC, Miura S, Tallquist MD, Behringer RR (2002). Generation of Bmpr/Alk3 conditional knockout mice. Genesis.

[24] Huang FW, Pinkus JL, Pinkus GS, Fleming MD, Andrews NC (2005). A mouse model of juvenile hemochromatosis. J. Clin. Invest..

[25] Gurieva I (2017). Erythropoietin administration increases splenic erythroferrone protein content and liver TMPRSS6 protein content in rats. Blood Cells, Mol. Dis..

