# Supplementary material for: The hemochromatosis protein HFE signals predominantly via the BMP type I receptor ALK3 in vivo
Source: Commun Biol. 2018 Jun 8;1:65. doi: 10.1038/s42003-018-0071-1 (PMC6123693; doi:10.1038/s42003-018-0071-1)
Supplement: Supplementary file 1 — Supplementary Information [file 42003_2018_71_MOESM1_ESM.pdf]

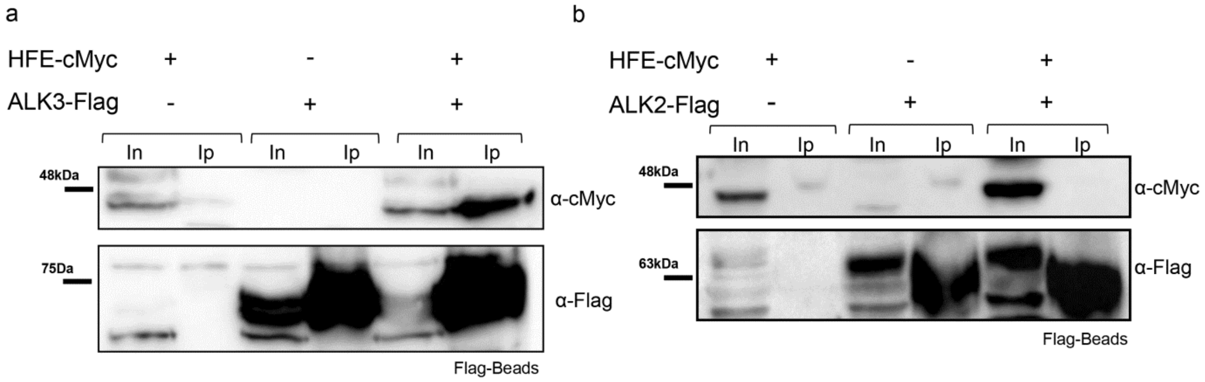

Supplementary Figure 1. (a) HFE interacts with ALK3 *in vitro*[6]. Huh7 cells were transfected with tagged receptors and co-immunoprecipitation was performed with Flag-Beads. Membranes were incubated with an antibody directed against cMyc and repobed with an antibody directed against Flag to test transfection and precipitation efficiency. In-Input; Ip- Immunoprecipitation (N=3). **(b) HFE and ALK2 did not interact i*n vitro*.** Huh7 cells were transfected with ALK2-Flag and HFE-cMyc and co-immunoprecipitation was performed with Flag-Beads. Membranes were incubated with an antibody directed against cMyc and reprobed with an antibody directed against Flag to test transfection and precipitation efficiency. In-Input; Ip-Immunoprecipitation (N=3).

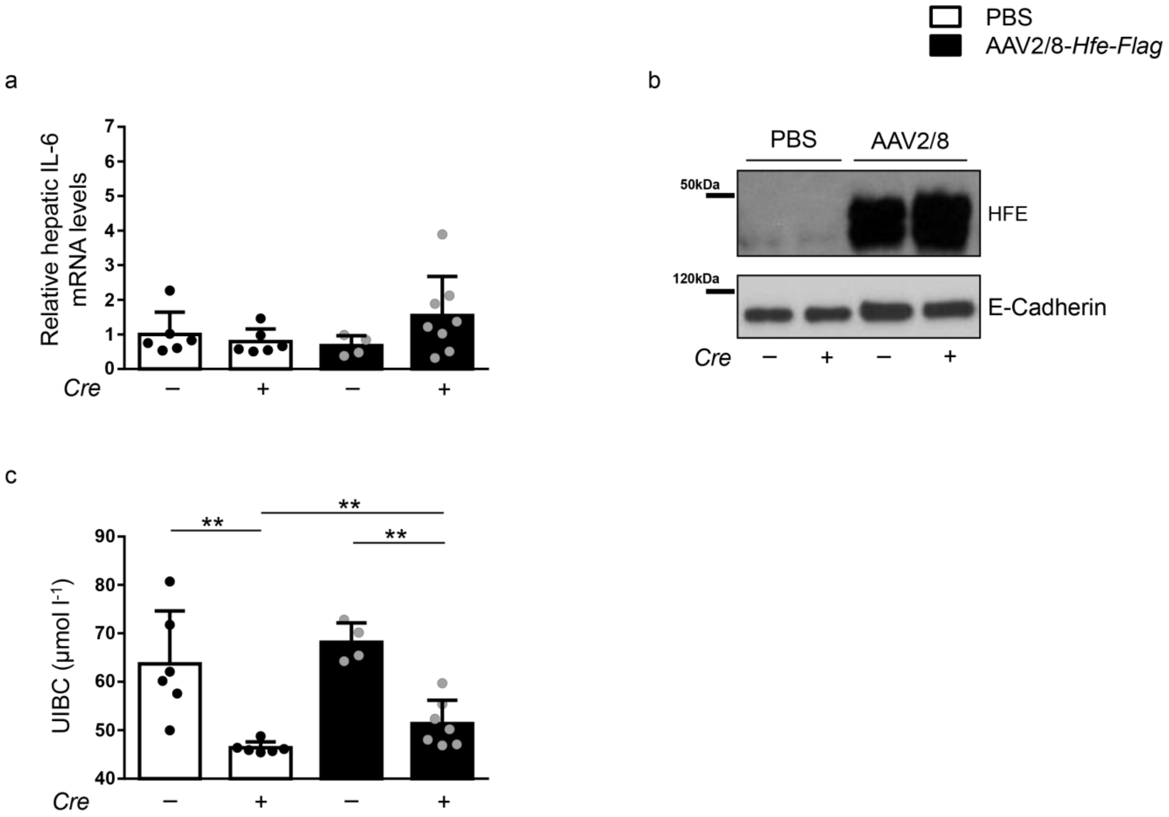

Supplementary Figure 2. (a) IL-6 mRNA levels did not increase after AAV2/8-*Hfe-Flag* administration. Hepatic IL-6 mRNA levels were determined to test whether virus application increases cytokine release and therefore hepcidin expression. IL-6 mRNA levels were not elevated after virus injection and were similar compared to PBS injected animals (*Alk3*$^{fl/fl}$*:* n=6; *Alk3*$^{fl/fl}$*; Alb-Cre*: n=6; *Alk3*$^{fl/fl}$ injected with AAV2/8-*Hfe-Flag*: n=4; *Alk3*$^{fl/fl}$*; Alb-Cre* injected with AAV2/8-*Hfe-Flag*: n=8). **(b) HFE protein levels in hepatic plasma membrane enriched fractions.** Representative samples of each genotype and each treatment on one immunoblot are shown. E-cadherin was used as a loading control. **(c) Overexpression of HFE increased UIBC.** Iron parameters were analyzed 14 days after virus injection. UIBC increased in both control and hepatocyte-specific *Alk3* deficient male mice injected with AAV2/8-*Hfe-Flag* compared to mice injected with (*Alk3*$^{fl/fl}$*:* n=6; *Alk3*$^{fl/fl}$*; Alb-Cre*: n=6; *Alk3*$^{fl/fl}$ injected with AAV2/8-*Hfe-Flag*: n=4; *Alk3*$^{fl/fl}$*; Alb-Cre* injected with AAV2/8-*Hfe-Flag*: n=7; **p≤0.008).

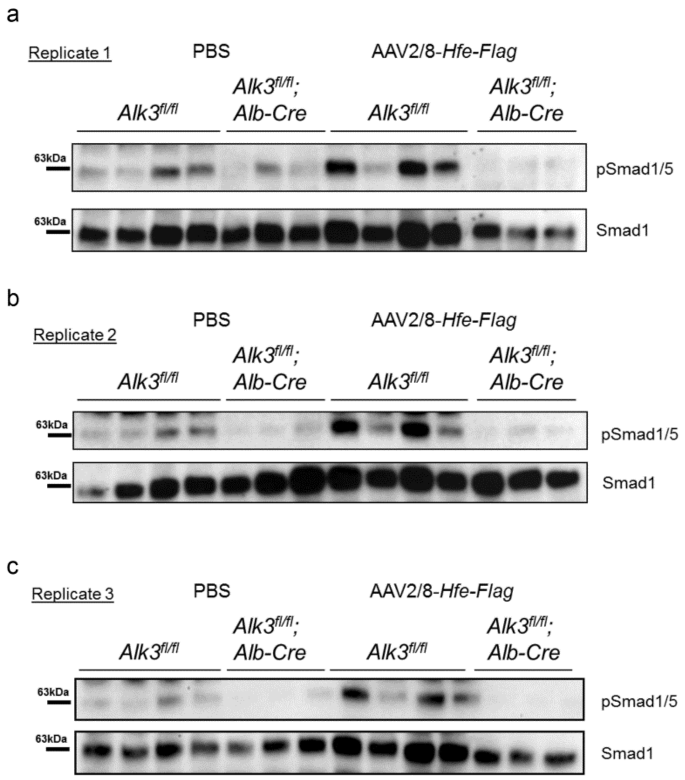

Supplementary Figure 3. Replicates of pSmad1/5 and Smad immunoblots. HFE overexpression increased pSmad1/5 levels in control mice, but not in mice with a hepatocyte-specific *Alk3* deficiency. **(a-c)** Hepatic phosphorylation of pSmad1/5 and total Smad1 protein levels in AAV2/8-*Hfe-Flag* or PBS injected mice with and without hepatocyte-specific *Alk3* deficiency are shown.

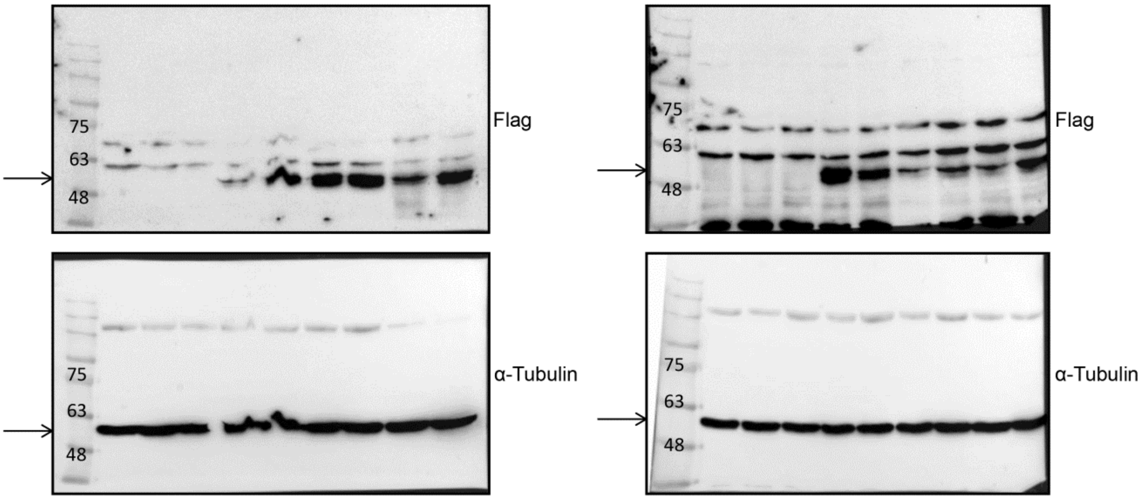

Supplementary Figure 4. Full uncropped and unedited versions of immunoblots depicted in Figure 1c.

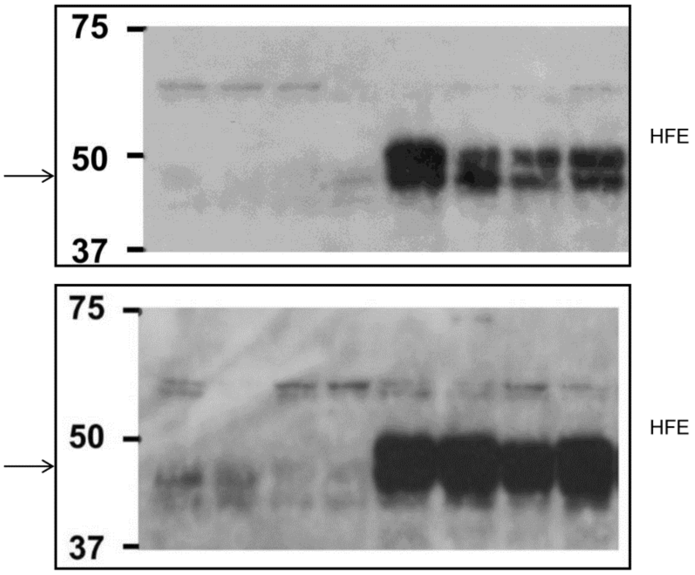

Supplementary Figure 5. Full uncropped and unedited versions of immunoblots depicted in Figure 1d.

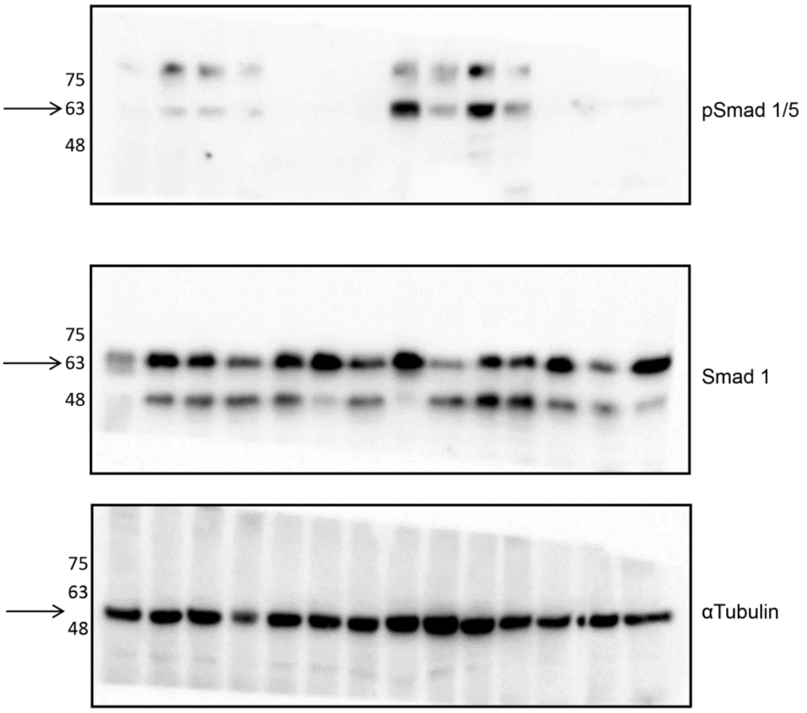

Supplementary Figure 6. Full uncropped and unedited versions of immunoblots depicted in Figure 5.

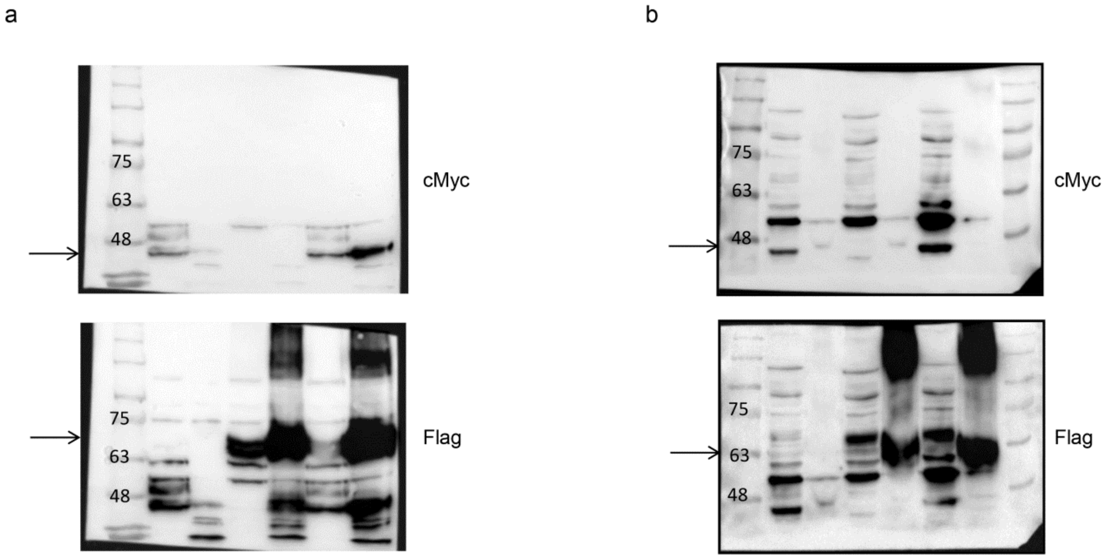

Supplementary Figure 7. Full uncropped and unedited versions of immunoblots depicted in Supplementary Figure 1a, b.

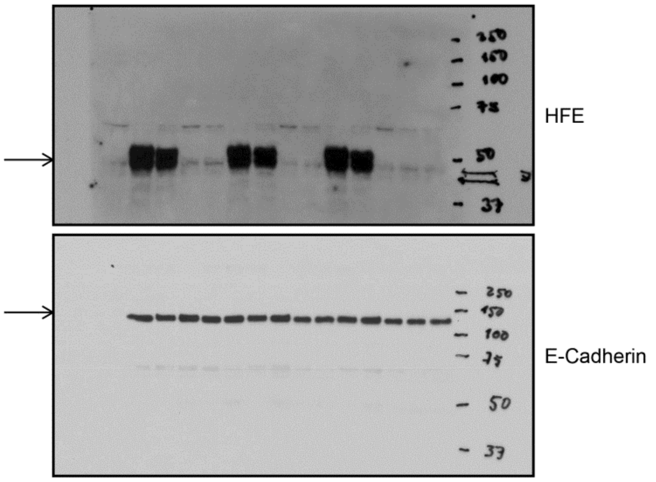

Supplementary Figure 8. Full uncropped and unedited versions of immunoblots depicted in Supplementary Figure 2b.

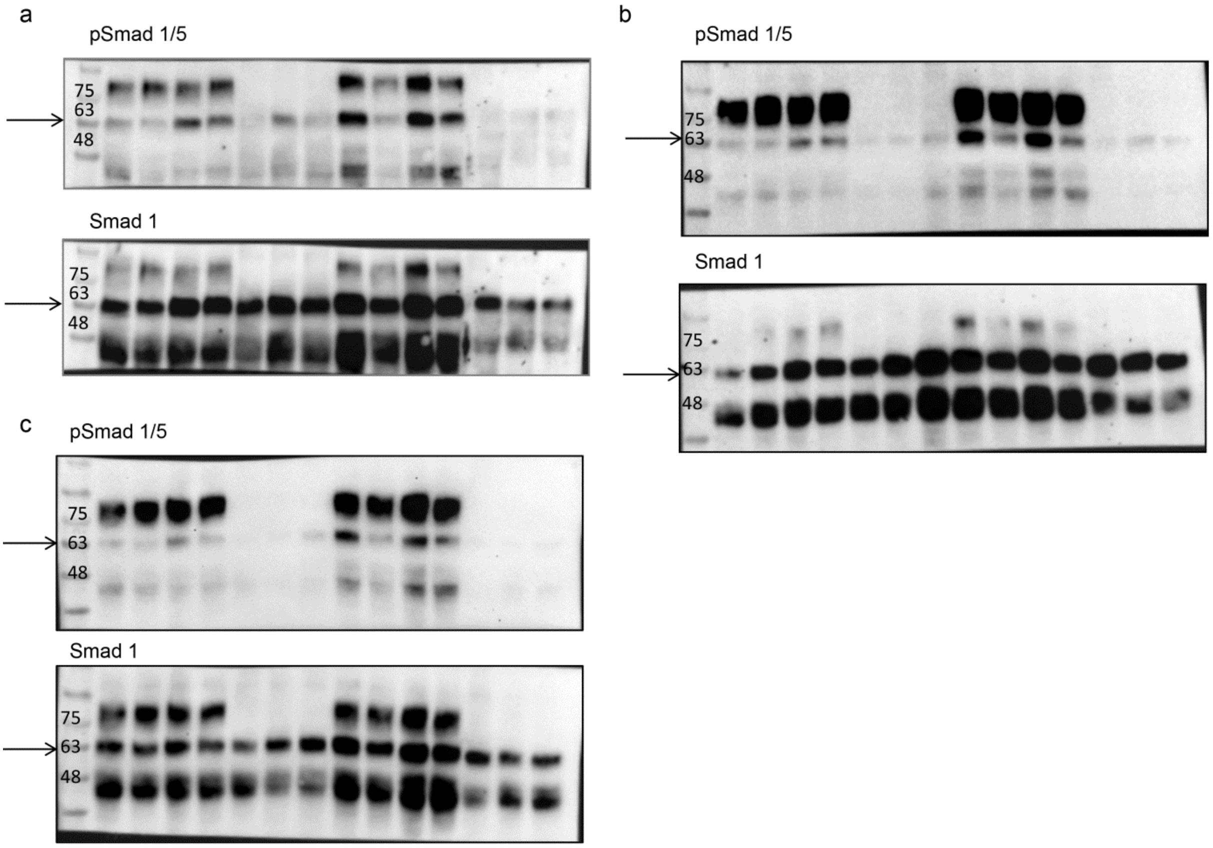

Supplementary Figure 9. Full uncropped and unedited versions of immunoblots depicted in Supplementary Figure 3a-c.

Supplementary Table 1. Semi quantitative real time PCR primer pairs

18S rRNA forward [1]	5'-CGGCTACCACTCCAAGGAA-3'
18S rRNA reverse	5'-GCTGGAATTACCGCGGCT-3'
Mouse Hepcidin forward [2]	5'-AAGCAGGGCAGACATTGCGAT-3'
Mouse Hepcidin reverse	5'-CAGGATGTGGCTCTAGGCTATGT-3'
Mouse HFE forward [3]	5'-CAGCTGAAACGGCTCCTG-3'
Mouse HFE reverse	5'-CGAGTCACTTTCACCAAAGTAGG-3'
Mouse IL-6 forward [4]	5'-AACGATGATGCACTTGCAGA-3'
Mouse IL-6 reverse	5'-TGGTACTCCAGAAGACCAGAGG-3'
Mouse ID1 forwards [1]	5'-TCTGTCGGAGCAAAGCGTGGCC-3'
Mouse ID1 reverse	5'-CCGGTGGTCCCGACTTCAGACT-3'
Mouse BMP-6 forward [5]	5'-ATGGCAGGACTGGATCATTGC-3'
Mouse BMP-6 reverse	5'-CCATCACAGTAGTTGGCAGCG-3'
Mouse ALK3 TaqMan Gene expression assay	Bmpr1a- Mm00477650_m1 Bmpr1a
Mouse S18 TaqMan Gene expression assay	RN 18S-Mm03928990_g1

Supplementary References

1. Steinbicker, A. U. *et al*. Perturbation of hepcidin expression by BMP type I receptor deletion induces iron overload in mice. *Blood* **118**, 4224-4230 (2011).

2. Steinbicker, A. U. *et al*. Inhibition of bone morphogenetic protein signaling attenuates anemia associated with inflammation. *Blood* **117**, 4915-4923 (2011).

3. Delima, R. D. *et al*. Disruption of hemochromatosis protein and transferrin receptor 2 causes iron-induced liver injury in mice. *Hepatology* **56**, 585-593 (2012).

4. Cui, W., Taub, D. D. & Gardner, K. qPrimerDepot: a primer database for quantitative real time PCR. *Nucleic Acids Res.* **35**, D805-9 (2007).

5. Kautz, L. *et al*. Iron regulates phosphorylation of Smad1/5/8 and gene expression of Bmp6, Smad7, Id1, and Atoh8 in the mouse liver. *Blood* **112**, 1503-1509 (2008).

6. Wu, X. G. *et al*. HFE interacts with the BMP type I receptor ALK3 to regulate hepcidin expression. *Blood* **124**, 1335-1343 (2014).
